# The transforming role of wharton’s jelly mesenchymal stem cell-derived exosomes for diabetic foot ulcer healing: a randomized controlled clinical trial

**DOI:** 10.1186/s13287-025-04690-y

**Published:** 2025-10-13

**Authors:** Mohamed S. Kishta, A. M. Hafez, Tamer Hydara, Zeinab Hamed, Mohamed M. Bahr, Ashraf A. Shamaa, Ahmed N. Abdallah

**Affiliations:** 1 Hormones Department, Medical Research and Clinical Studies Institute, National Research Centre, Dokki, 12622 Cairo Egypt; 2 Stem Cell Lab., Center of Excellence for Advanced Sciences, National Research Centre, Dokki, 12622 Cairo Egypt; 3 Medical Biochemistry Department Faculty of medicine, Kafr Elsheikh University, Kafr El-Sheikh, Egypt; 4 Department of Internal Medicine, Faculty of Medicine, Kafrelsheikh University, Kafrelsheikh, 33516 Egypt; 5 Surgery, Anesthesiology and Radiology department, Faculty of Veterinary, Medicine Cairo University, Cairo, Egypt; 6 Surgery, Anesthesiology and Radiology dept, Faculty of Veterinary Medicine, Cairo University, Cairo, Egypt

## Abstract

**Background:**

Diabetic foot ulcers (DFUs), which have high rates of recurrence, amputation, and death, are a significant complication in the therapy of diabetes. Chronic inflammation, vascular dysfunction, and peripheral neuropathy are the results of their etiology, which includes dysregulated glucose homeostasis. These elements contribute to the poor clinical outcomes of DFUs and their complexity. Exosomes, which are natural nanovesicles that promote intercellular communication by transporting functional molecular cargos such as proteins, lipids, and nucleic acids, are being investigated as novel treatment approaches for diabetic foot ulcers (DFUs). These exosomes present a viable therapy option for DFU because they can alter cellular functions and promote wound healing.

**Methods:**

To improve wound healing in patients with diabetic foot ulcers (DFUs), we assessed the safety and effectiveness of Wharton’s jelly-derived mesenchymal stem cell (WJ-MSC) exosomes in this study. 110 individuals with persistent DFUs participated in our research. Three groups were randomly selected from among the participants. For 4 weeks, the first group got weekly topical application of WJ-MSC exosome along with standard of care (SOC); the second control group received SOC alone; and the third placebo group received SOC together with CMC (the exosome vehicle). While effectiveness outcomes comprised the rate of wound closure and the duration to full epithelialization, safety endpoints included the frequency of adverse events.

**Results:**

According to our study’s findings, 53 patients (62%) had fully recovered by the end of the study, and the treated group had a significantly higher percentage of patients who had fully recovered than the control group. The treated group’s mean time to fully recover was 6 weeks (range: 4–8 weeks), while the controls were 20 weeks (range: 12–28 weeks).

**Conclusions:**

Our research proved that MSC-Exos is a viable treatment option for DFUs. MSC-Exos provide a multimodal approach to improve wound healing outcomes in diabetes patients.

## Introduction

One of the deadliest chronic illnesses in the world is diabetes. It’s predicted global prevalence was 10.5% in 2019 (536.6 million people), and by 2045, it is expected to rise to 12.2% (783.2 million people). Diabetic foot ulcers (DFU), which cause 85% of diabetic lower-limb amputations (DLLA), are one of its worst side effects [1, 2]. Significant mortality and high recurrence rates are other characteristics of DFU; more than 40% of afflicted individuals pass away within 5 years [3].

Diabetic foot ulcers (DFUs) are a significant complication associated with type 2 diabetes mellitus (T2DM), posing a major risk for non-traumatic lower limb amputations. These ulcers are prevalent among diabetic patients and are linked to chronic vascular complications such as diabetic kidney disease and retinopathy [4].

Diabetic foot ulcers (DFUs) are among the most severe chronic complications of diabetes, significantly impairing patients’ quality of life and contributing to high rates of disability and amputation [5]. Current treatment strategies focus on improving blood flow, controlling infection with antibiotics, and utilizing specialized wound-healing dressings [6]. However, the effectiveness of these approaches is often limited, particularly in advanced cases, as they fail to address the underlying biological mechanisms that delay healing.

In recent years, researchers have begun to explore novel techniques involving stem cells and extracellular vesicles (exosomes) as innovative solutions for treating chronic wounds [7]. Extracellular vesicles derived from Wharton’s Jelly mesenchymal stem cells (WJ-MSCs) are particularly promising due to their ability to deliver active growth factors, proteins, and genetic material that promote natural wound healing processes [8].

The chronic nature of DFUs and the intricate interactions between variables that hinder healing, such as infection, neuropathy, and poor blood circulation, make care of these conditions extremely difficult and increase the risk of amputations [9]. Numerous strategies, including the use of growth hormones or skin substitutes, unloading methods, and sophisticated wound dressings, have been developed to promote healing and avoid recurrence [10]. But they are still far from ideal. As such, there is an urgent need for innovative therapeutic strategies that can effectively promote wound healing and improve patient outcomes [11].

Exosomes generated from mesenchymal stem cells (MSCs) have attracted interest in regenerative medicine in recent years. Exosomes are tiny extracellular vesicles that carry proteins, lipids, and nucleic acids, therefore promoting intercellular communication [12]. Exosomes generated from MSCs have shown promise in reducing inflammation, fostering angiogenesis, and improving tissue healing in several ailments, such as chronic wounds, osteoarthritis, and cardiovascular disorders [13].

Wharton’s jelly mesenchymal stem cells (WJ-MSCs), which are formed from Wharton’s jelly (WJ), are distinct from other sources of umbilical cord-derived MSCs. WJ-MSCs have several benefits, including a high potential for differentiation and immune purification, and are very accessible and morally uncontroversial [14]. Additionally, they have traits with embryonic stem cells, such as strong expansion potential and rapid cell division [15].

The numerous benefits of WJ-MSC exosomes offer promise for the treatment of DFU. These exosomes demonstrated that WJ-MSC exosomes efficiently stimulate keratinocyte migration and proliferation, presumably because of their increased fibrinogen beta chain (FGB) concentration. A crucial component of fibrinogen, FGB may be transformed into fibrin and act as a temporary extracellular matrix (ECM) during wound healing, offering structural support for keratinocyte migration and adhesion [16].

Furthermore, MSC-exosomes have demonstrated a potent anti-inflammatory function; they may enhance M2 polarization over M1 by regulating inflammatory cytokine levels, which includes downregulating pro-inflammatory mediators like TNF-α and IL-1β and upregulating the anti-inflammatory cytokine IL-10. Additionally, by upregulating the expression of Foxp3 (a crucial transcription factor for the formation and function of regulatory T cells) and IDO, exosomes aid in T cell differentiation and encourage the transition toward anti-inflammatory phenotypes, such as regulatory T (Treg) cells. An essential component of immunological tolerance, IDO is an enzyme involved in the metabolism of tryptophan. In the context of immune suppression or control, it is frequently increased in immune cells such as dendritic cells and other antigen-presenting cells. A vital amino acid called tryptophan is broken down by IDO, which results in tryptophan depletion and the buildup of harmful metabolites like kynurenine. This encourages the development of regulatory T cells (Tregs) and suppresses T cell proliferation [17].

Regarding vascularization, exosome-mediated delivery of HGF (Hepatocyte growth factor) supports vascular stability and enhances neovascularization by activating the PTEN/PI3K/Akt and MAPK signaling pathways [18,19]. Human umbilical cord mesenchymal stem cell-derived exosomes (hUCMSC-Exos) are enriched in miR-21, miR-23a, miR-125b, and miR-145, a specific group of miRNAs that inhibit myofibroblast activation and attenuate actin production and collagen deposition results in reduced scar formation and improved tissue remodeling in late stages of wound healing [20].

In conclusion, WJ-MSC generated exosomes are believed to have considerable promise for diabetic foot ulcer healing, working almost at every stage of wound healing, from decreased inflammation to improved neovascularization and epithelization to decreased scar risk through tissue remodeling.

To explore a potential new therapeutic paradigm for this crippling illness, we set out to determine the safety and effectiveness of topical administration of WJ-MSC-derived exosomes in patients with chronic DFUs. In addition to increasing healing rates, the research’s findings may greatly raise the standard of living for those with diabetic foot ulcers.

## Methods

### Isolation and characterization of WJ-MSC cells from UC

After gaining informed consent, the university hospital used aseptic surgery to remove the umbilical cord tissue from a healthy donor. Before dissecting Wharton jelly (WJ), UC tissue was submerged in phosphate-buffered saline (PBS) containing 100 U/ml penicillin, 100 µg/ml streptomycin, and 2 µg/ml amphotericin B [21]. WJ was centrifuged at 340xg after being treated for an hour at 37 °C with collagenase (1 mg/ml type I) and hyaluronidase (0.7 mg/ml). The cell pellet was mixed with DMEM/F12 supplemented with 15% FBS and incubated at 37 °C with 5% CO_2_. Fresh media was added to the cells every 4 days throughout the 21-day observation period [22]. WJ-MSC cells’ morphology was examined under a microscope. During the culture period, the medium was replaced every 3–4 days to ensure proper cell growth. Cells were split at a 1:4 ratio upon reaching confluence to maintain adequate growth conditions and prevent overcrowding. In WJ-MSC cells during the 21st-day passage, flow cytometric examination revealed the presence of CD14, CD34, CD73, and CD105 labeling. WJ-MSC cell surface receptors CD14, CD34, CD73, and CD105 staining were subjected to the immunofluorescence technique [23].

### Isolation and characterization of exosomes from WJ-MSC

For 48 h, MSC cells were cultured in 75 cm^2^ flasks using DMEM/F12 mix that was devoid of FBS (starved). Exosomes secreted from fasting cells during a 48-h period were first obtained by collecting the media. To separate the cells and big vesicles, the fluids were centrifuged for 10 min at 13,000×*g* and 10 min at 45,000×*g*. After that, it was centrifuged in an ultracentrifuge for 5 h at 110,000×*g* (Beckman Coulter) [24]. Ultimately, the pellet was suspended in PBS and the supernatant was discarded [25]. Using flow cytometry, the characteristics of the isolated exosomes were examined for the CD9, CD63, CD81, and HSP70 markers. WJMSC exosomes were incubated with 1.5 × 10^5^ anti-CD63 beads in 50 ml PBS at room temperature for 15 min to perform a flow cytometric analysis. The beads were incubated for the entire night at 4uC with mild stirring after the volume was increased to 300 ml. After 30 min of incubation in 100 mM glycine, the process was halted [26]. Following two rounds of washing, exosome-coated beads were incubated in 50 mg of human IgG (Sigma-Aldrich) for 15 min at 4 °C. They were then treated with anti-CD9 FITC, anti-CD63 PE, anti-CD81 APC, or matched isotype controls (BD Biosciences) and obtained using a FACS Melody (BD Biosciences) [27]. TEM electron microscopy was used to determine the morphology and nano-size of WJMSCs exosomes [28]. The isolation and characterization process were repeated three times to confirm reproducibility and consistency in results.

### Study design

To assess the effectiveness and safety of WJ-MSC-derived exosome gel in patients with diabetic foot ulcers (DFUs), a randomized double-blind controlled clinical experiment was carried out. The trial protocol was implemented after obtaining the Scientific Research Ethics Committee of Kafr Elsheikh University on 25/3/2024, with final approval by the Committee’s decision No. KESIRB200-175. With Clinical Trial ID. NCT06812637 on ClinicalTrials.gov https://clinicaltrials.gov/study/NCT06812637. All participants provided written informed consent before enrollment.

#### Participants

After 207 patients were assessed, 110 of them satisfied the requirements for inclusion. The patients were then split into three groups:

- Group treated: 40 patients received standard of care (SOC) once weekly for 4 weeks, followed by a 16-week follow-up, using Wharton jelly derived mesenchymal stem cell (WJ-MSC) exosome gel [29].
- Control group: 35 patients had just standard of care (SOC) for 4 weeks, then 16 weeks of follow-up.
- A visually similar saline-based formulation was administered once weekly to 35 patients in the placebo group for 4 weeks, followed by follow-up for 16 weeks, along with SOC [29].

**Analysis After Dropouts**:

- Group treated (SOC + WJ-MSC-derived exosome gel):
  - 30 patients completed the pre-protocol analysis.
  - Dropouts included: 3 withdrew consent, 1 missed 6 consecutive dressings, 5 were lost to follow-up, and 1 developed osteomyelitis.
- Control Group (SOC only):
  - 24 patients completed the pre-protocol analysis.
  - Dropouts included: 5 withdrew consent, 2 missed 6 consecutive dressings, 2 were lost to follow-up, and 2 developed osteomyelitis.
- Placebo Group (SOC with vehicle):
  - 31 patients completed the pre-protocol analysis.
  - Dropouts included: 1 death, 1 amputation,1 was lost to follow-up, and 1 developed osteomyelitis.

**Fig. 1:**
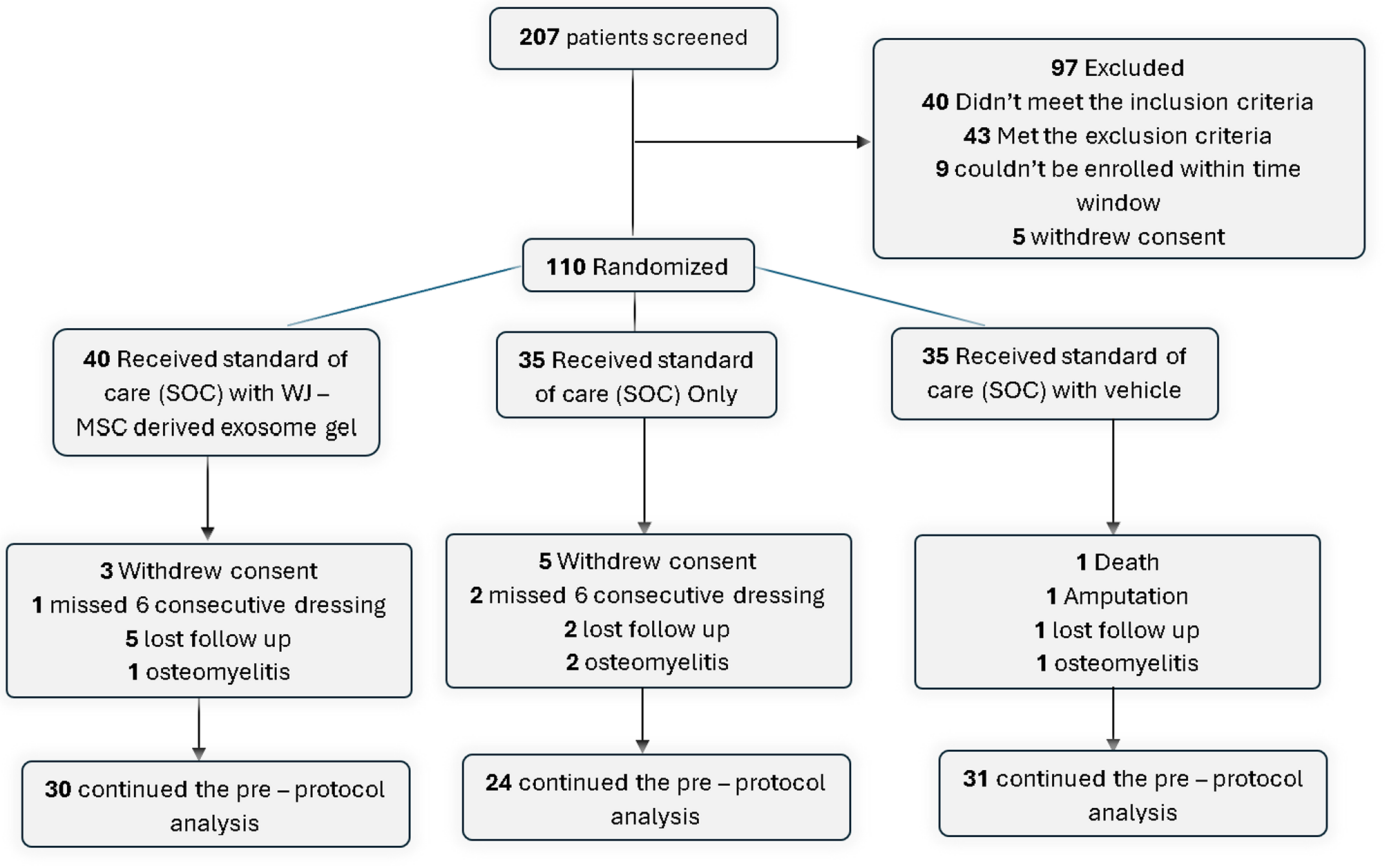
Consort flow diagram showing the progress through the phases of the trial

A consort flow diagram showing the progress through the trial phases is shown in Fig. 1.

#### Inclusion criteria

1. People with type 2 diabetes who are 42–62 years of age.
2. The existence of a persistent DFU that does not go away after 7 days of standard of care (SOC) treatment or that does not shrink by more than 30%.
3. Ulcers smaller than 30 cm² that are seen on the plantar, medial, or lateral portions of the foot.
4. Individuals suffering from ischemic, neuropathic, or mixed neuropathic-ischemic ulcers.
5. Revascularization performed for ischemic ulcers before enrollment.

#### Exclusion criteria

1. Pregnancy or breastfeeding.
2. Type 1 diabetes who are 18 years of age or older.
3. Presence of venous ulcers or active infections.
4. Exposure of bone, ligaments, or tendons.

Demographic data, comorbidities, and concomitant medications were recorded. All participants received instructions on ulcer care and offloading.

### Methods of evaluation of ulcer treatment outcome

#### Classification of ulcer

Two systems were used for classification: the Wound, Ischemia, and Foot Infection system (WIFI system) and the Site, Ischemia, Neuropathy, Bacterial Infection, Area, Depth system (SINBAD system) [30].

##### The SINBAD

The SINBAD method makes it easier to classify diabetic foot ulcers (DFUs) using a 0–1 point scale. The severity of the ulcer is indicated by a score that ranges from 0 to 6 [31]. This approach is easy to use, gives good intra-observer and modest inter-observer repeatability, and doesn’t require any specific equipment beyond standard clinical examination. The SINBAD system is a useful tool for clinical application, as recommended by the IWGDF, and it efficiently monitors ulcer progression, healing, and amputation risk Table 1.

**Table 1:** SINBAD system

Category	Definition	Score
Site	Forefoot	0
	Midfoot and hindfoot	1
Ischemia	Pedal blood flow intact: At least one palpable pulse	0
	Clinical evidence of reduced pedal flow	1
Neuropathy	Protective sensation intact	0
	Protective sensation lost	1
Bacterial infection	None	0
	Present	1
Area	Ulcer < 1 cm^2^	0
	Ulcer ≥ 1 cm^2^	1
Depth	Ulcer confined to skin and subcutaneous tissue	0
	Ulcer reaching muscle, tendon or deeper	1
Total possible score		6

##### The WIFI system

The WIFI system, developed by the Society for Vascular Surgery in 2014, addresses the limitations of existing classifications by evaluating three major risk factors for amputation: wound characteristics, ischemia (based on ABI scores), and infection. Each factor is scored from 0 to 3, providing a detailed severity assessment as shown in Table 2. While the WIFI system aids in predicting major amputations and guiding interventions like revascularization, its reliance on specialized vascular measurements limits its utility in primary or community care settings, making it more suitable for specialized vascular clinics.

**Table 2:** WIFl system

Grade	Wound	Ischemia			Foot infection system
	Clinical features	ABI (mmHg)	ASP (mmHg)	Toe pressure, TcPO_2_ (mmHg)	Clinical manifestations
0	No ulcer no gangrene	≥ 0.80	> 100	≥ 60	no infection-related symptoms or indicators. At least two of the following symptoms indicate the presence of an infection: (1) localized induration or swelling; (2) 0.5–2 cm of erythema surrounding the ulcer; (3) localized pain or soreness; (4) localized warmth; and (5) purulent discharge (thick, opaque to white, or bloody fluid).
1	No exposed bone, except restricted to the distal phalanx, and one or more small, shallow ulcers on the distal leg or foot	0.6–0.79	70–100	40–59	Additional factors that might trigger an inflammatory reaction in the skin include trauma, gout, acute When a local infection just affects the skin and subcutaneous tissue, it is eliminated along with Charcot neuro-osteoarthropathy, fracture, thrombosis, and venous stasis.
2	Shallow heel ulcer without calcaneal involvement, gangrenous alterations restricted to digits; deeper ulcer with exposed bone, joint, or tendon, usually without affecting the heel	0.4–0.59	50–70	30–39	Local infection with erythema more than 2 cm or affecting subcutaneous tissues and structures deeper than the skin (such as fasciitis, septic arthritis, osteomyelitis, or abscess) without any indications of a systemic inflammatory response
**3**	Full-thickness heel necrosis with calcaneal involvement; deep, full-thickness heel ulcers with or without calcaneal involvement; severe gangrene including the forefoot and/or midfoot; and extensive, deep ulcers involving the forefoot and/or midfoot	≥ 0.39	< 50	< 30	Local infection with signs of SIRS, as manifested by two or more of the following: (1) Temperature > 38 °C or < 36 °C; (2) Heart rate > 90 beats/min; (3) Respiratory rate > 20 breaths/min or PaCO_2_ < 32 mmHg; and (4) White blood cell count > 12,000 or < 4000 cu/mm or 10% immature bands

##### To classify foot infection, we use the international working group on the diabetic foot (IWGDF)/Infectious diseases society of America (IDSA system) [32]

The categorization method is used to determine whether a diabetic has a foot infection and how serious it is, as shown in Table 3.

**Table 3:** IWGDF-IDSA system

Clinical classification of infection, definitions	IWGDF/IDSA classification
No systemic or local symptoms or signs of infection	1/Uninfected
Infected: At least two of these items are present: • Local swelling or induration • Erythema > 0.5 but < 2 cmb around the wound • Local tenderness or pain • Local increased warmth • Purulent discharge	2/Mild
And no other cause of an inflammatory response of the skin (e.g., trauma, gout, acute charcot neuro-arthropathy, fracture, thrombosis, or venous stasis)	
Infection with no systemic manifestations and involving: • Erythema extending ≥ 2 cmb from the wound margin, *and/or* • Tissue deeper than skin and subcutaneous tissues (e.g., tendon, muscle, joint, and bone).	3/Moderate
Infection involving bone (osteomyelitis)	Add “(O)”
Any foot infection with associated systemic manifestations (of the systemic inflammatory response syndrome [SIRS]), as manifested by ≥ 2 of the following: • Temperature, > 38 °C or < 36 °C • Heart rate, > 90 beats/min • Respiratory rate, > 20 breaths/min, *or* PaCO2 < 4.3 kPa (32 mmHg) • White blood cell count > 12,000/mm^3^, *or* < 4G/L, *or* >10% immature (band) forms	4/Severe
- Infection involving bone (osteomyelitis)	Add “(O)”

The presence of clinically significant foot ischemia makes both diagnosis and treatment of infection considerably more difficult.

- Infection refers to any part of the foot.
- In any direction, from the rim of the wound.
- If osteomyelitis is demonstrated in the absence of ≥ 2 signs/symptoms of local or systemic inflammation, classify the foot as either grade 3(O) (if < 2 SIRS criteria) or grade 4(O) if ≥ 2 SIRS criteria).

#### Assessment of ulcer healing outcome clinically

An Android smartphone was used to measure the ulcer’s length, breadth, and surface area exactly [33]. The camera was positioned 25 cm from the ulcer, making sure it was parallel to the wound. After taking the picture, the operator marked the edges of the wound and determined the size of the ulcer. A blinded medical practitioner assessed each wound three times to ensure reproducibility, and statistical analysis was performed using the average of these data [29]. Ulcer size reduction will be computed using the following formula: ulcer size reduction = (Ai – Af)/Ai × 100, where Ai is the initial ulcer area and Af is the ulcer area during follow-up after treatment. Ulcers were assessed and photographed for healing [34].

#### Management of ulcer and application of WJ-MSC exosome gel

Debridement of diabetic foot ulcers was initially performed to remove hyperkeratotic skin or necrotic and infected tissues. Following that, the area was cleaned with regular saline. Before beginning any research, measurements were made of the ulcer’s length, width, and surface. After applying the Wharton jelly derived mesenchymal stem cell (WJ-MSC) exosome gel to the ulcer, the treatment group covered the region with sterile gauze and a non-compressible bandage. After a month of doing every 3 days, the wound was irrigated with regular saline, examined for infection, and then treated with Wharton jelly derived mesenchymal stem cell (WJ-MSC) exosome gel. The SOC was administered to the control and placebo groups, which included removing necrotic, hyperkeratotic, and infected tissues, washing the wound with regular saline, and covering the ulcer with non-compressible bandages and sterile gauze. The patients were instructed to change the bandages every day and wipe the ulcer with regular saline [29].

##### Follow-up

Participants were followed up for 16 weeks with evaluations conducted at 2, 4 weeks, then 6 weeks, and then at 2, 4, and 6 months [29]. At each visit:

1. Ulcers were cleansed and assessed for infection.
2. A record of the interim medical history was kept, which included adverse events and prescription drugs.
3. Photographs of the ulcers were taken at each time point.

##### Endpoints

**Primary Endpoint**:

- The percentage of ulcer size decreases at 16 weeks.

**Secondary Endpoints**:

- The average reduction in ulcer size throughout the research.
- Complete healing rate (100% re-epithelialization without drainage).
- Safety assessment, including adverse events and tolerability [29].

##### Evaluation of outcomes

The study utilized clinical classification systems, including the SINBAD and WIFI systems, to stratify ulcers by severity, vascular health, and infection risk. Evaluating an ulcer involves several diagnostic tools to uncover underlying complications. To provide a comprehensive picture of systemic health and inflammation, laboratory tests usually include fasting blood sugar levels, glycated hemoglobin (HbA1c), a full metabolic panel, complete blood count (CBC), erythrocyte sedimentation rate (ESR), and C-reactive protein (CRP).

Imaging studies play a critical role, with plain X-rays used to identify hidden issues such as osteomyelitis, subcutaneous air, fractures, or foreign bodies. When osteomyelitis is suspected, MRI stands out as the preferred diagnostic tool due to its superior accuracy. Peripheral vascular disease can be assessed using arterial Doppler and ankle-brachial index (ABI) measurements, ensuring vascular factors are addressed. The probe-to-bone (PTB) test provides a straightforward but efficient way to identify osteomyelitis at the patient’s bedside: if a sterile metal probe contacts bone while exploring the ulcer, the test is positive. When treating diabetic individuals who may have bone infections, this rapid and accurate test is quite helpful [35].

### All patients in the three groups mentioned above were subjected to

1. To get rid of necrotic or diseased tissues, wounds were debrided.
2. After cleaning the region with regular saline, the therapy was administered.
3. The ulcers were covered with non-compressive bandages and sterile gauze.
4. Participants were instructed to clean and redress ulcers daily.

### Statistical analysis

The non-parametric Wilcoxon rank-sum test was used to evaluate differences in continuous variables of interest, while the chi-square test (or Fisher’s exact, if more applicable) was employed to compare categorical variables between groups. The Wilcoxon signed-rank test was used for post hoc comparisons to find changes in ulcer area across the timepoints of interest, and the non-parametric Friedman test was used to find differences in ulcer size at baseline, 2 weeks, and 4 weeks for the effects of therapy. Software GraphPad Prism (GraphPad version 8.0.2) was used to conduct statistical analysis. When P was less than 0.05, statistical significance was reached.

## Results

### Stem cells characterization

**Fig. 2:**
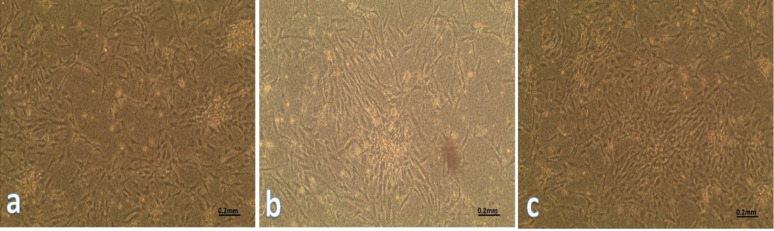
Morphological appearance of WJ-MSCs. **a** WJ-MSCs 3 days after isolation with confluency 10–20%, **b** WJ-MSCs 7 days after isolation with confluency 60–70%, and **c** WJ-MSCs 21 days after isolation with confluency 95% and complete sheet

Morphological appearance of WJ-MSCs was observed under an inverted microscope, cells typically exhibit a spindle-shaped morphology with a pronounced fibroblastic appearance. cells are arranged in a net Wharton jelly derived mesenchymal for ork, displaying prominent cytoplasmic extensions. Their uniform size and elongated shape indicate healthy proliferative potential as it was shown in Fig. 2a with confluency 10–20% after 3 days of isolation, Fig. 2b with confluency 60–70% after 7 days of isolation, and Fig. 2c showing complete sheet with confluency 95% after 21 days of isolation.

**Fig. 3:**
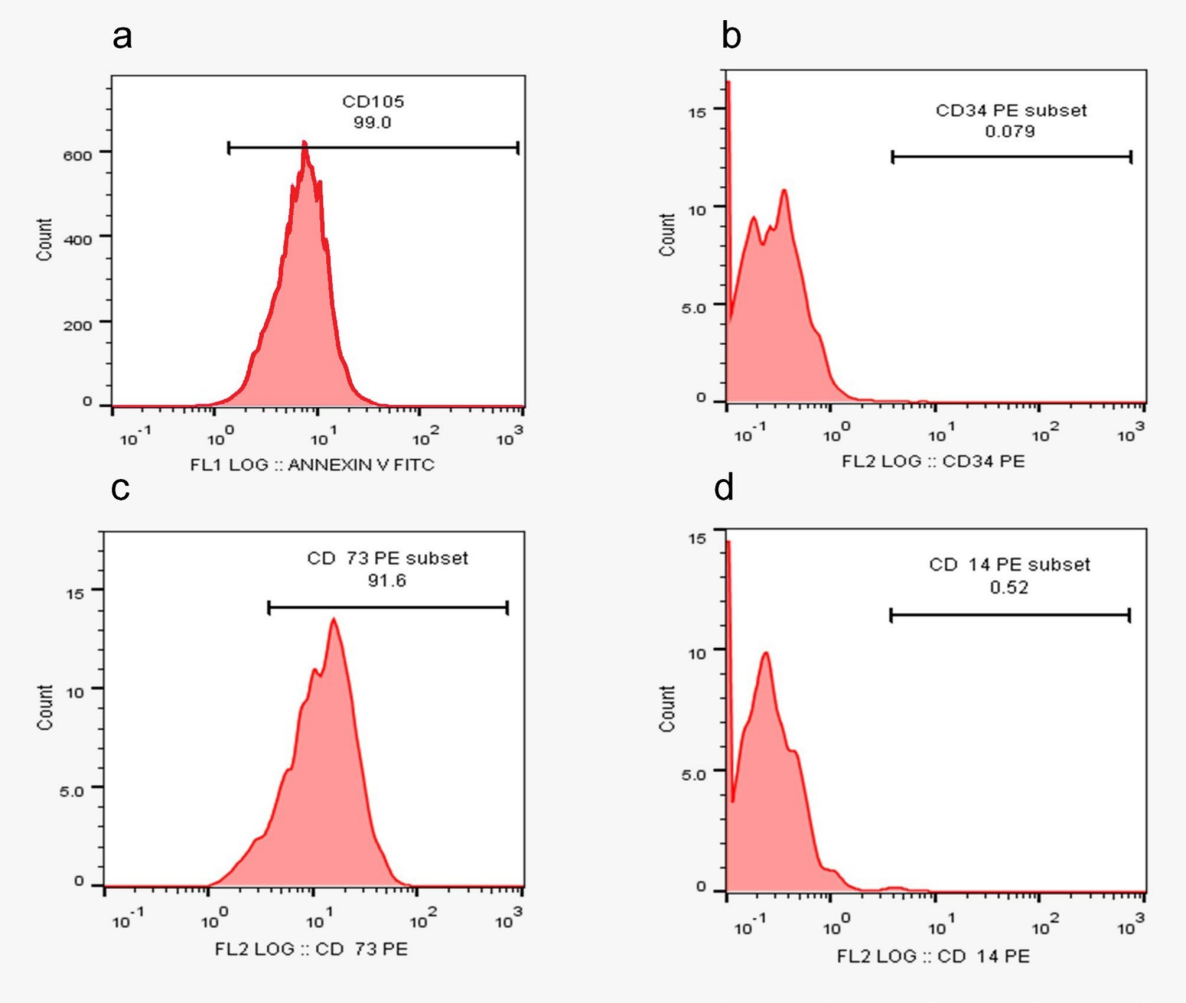
Characterization for the isolated cells by flow cytometric analysis of CD34, CD14, CD73 and CD105. Flow cytometry analysis showed high expression of CD105 (99%) in **a**, minimal CD34 (0.079%) in **b**, CD73 (91.6%) in **c**, and CD14 (0.52%) in **d**, indicating MSC identity

Also, the flow cytometric analysis for CD marker expression shown in Fig. 3. In Wharton’s jelly-derived mesenchymal stem cells (MSCs), the results indicate a high expression of mesenchymal markers CD73 (91.6%) and CD105 (99%), both of which are indicative of mesenchymal stem cell characteristics. However, the presence of CD14 (0.52%) and CD34 (0.079%) markers, which are typically absent in pure MSCs.

### WJ-MSC EVs characterization

**Fig. 4:**
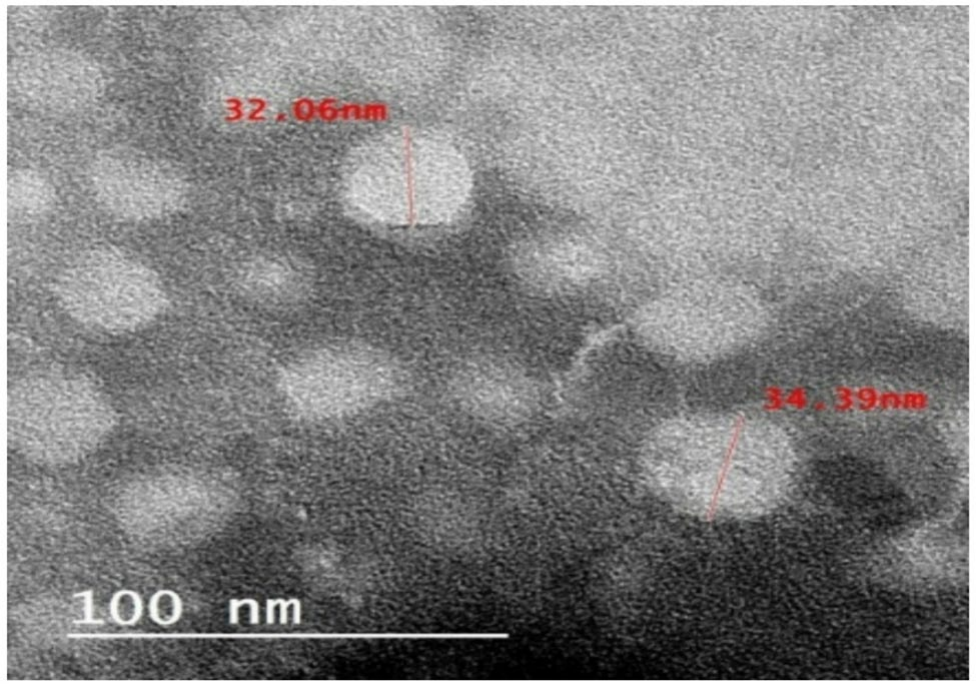
TEM image displays spherical, lipid bilayer-bound vesicles with sizes ranging from approximately 32 nm to 34 nm, as indicated by the scale bar (100 nm). These dimensions are on the lower end of the typical exosome size range (30–150 nm), suggesting successful isolation of small extracellular vesicles (sEVs)

**Fig. 5:**
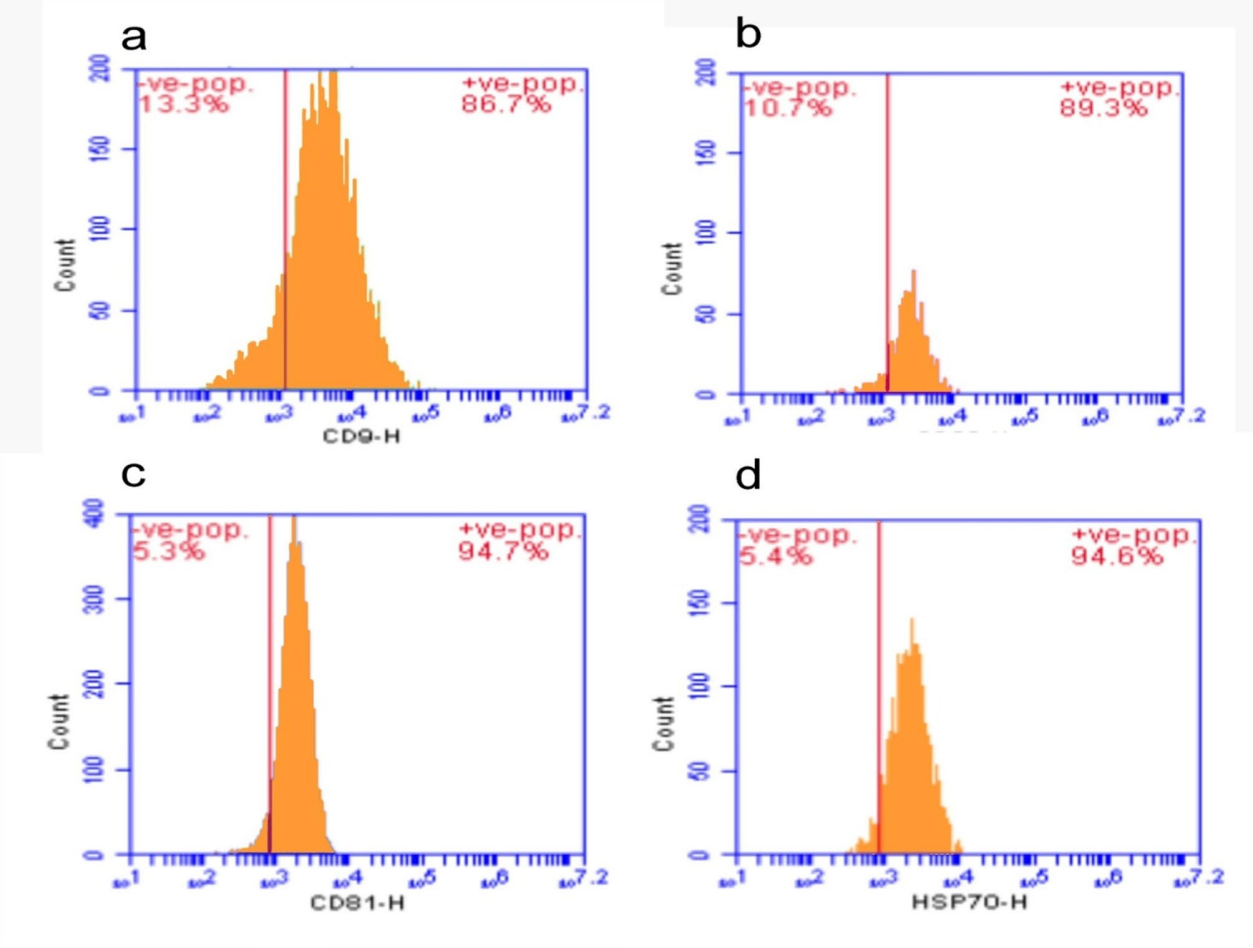
Characterization of exosomes showed that exosomes were positive for CD9, CD63, CD81, and HSP70. **a** show the CD9 with a positive value of 86.7%, **b** show the CD63 with a positive value of 89.3%, **c** show the CD81 with a positive value of 94.7%, and **d** show the HSP70 with a positive value of 94.6%

First, EV samples were measured via transmission electron microscope (TEM)as shown in Fig. 4. Flow cytometry analysis of exosomes was performed to detect the CD surface markers CD9, CD63, CD81, and HSP70 [36, 37]. The exosomes showed positivity to all CD markers as shown in Fig. 5.

### Patient assessment and evaluation of ulcer healing

**Fig. 6:**
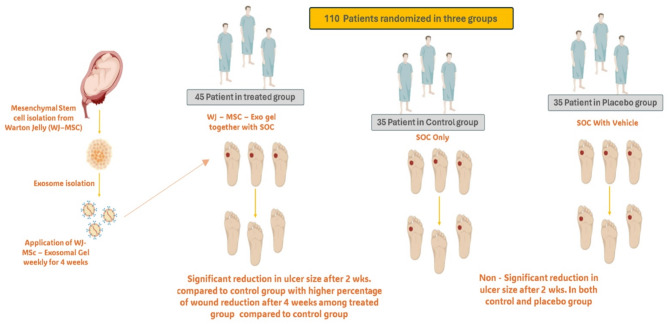
Schematic presentation of different groups in the study and overall outcome**s.** a clinical trial involving 110 patients divided into three groups to evaluate the effects of a gel derived from mesenchymal stem cells isolated from Wharton’s Jelly (WJ-MSC) with exosomes. The treated group (45 patients) received WJ-MSC exosomes gel alongside the standard of care (SOC) weekly for 4 weeks, showing a significant reduction in ulcer size after 2 weeks and substantial wound reduction after 4 weeks compared to the control group. The control group (35 patients) received only SOC, while the placebo group (35 patients) received SOC with a vehicle, neither of which demonstrated a significant reduction in ulcer size after 2 weeks

110 patients in all fulfilled the study’s inclusion and exclusion requirements. They were divided into three groups at random: the treatment group (*n* = 40), the control group (*n* = 35), and the placebo group (*n* = 35). An overall total of 85 patients—30 in the treatment group, 24 in the control group, and 31 in the placebo group—finished the trial and were included in the final analysis Fig. 6. Table 4. displays baseline ulcer features, type, medical state, and demographic information. The treated group’s mean age was 52 *±* 8 (range 44–62), and there was no discernible age difference between the groups (Wilcoxon rank-sum *P* = 0.571) and sex (Chi-square *P* = 0.77). HbA1c (Wilcoxon rank-sum *p* = 0.845) and the length of diabetes (Chi-square *p* = 0.21) did not differ statistically significantly. Approximately 36% of all participants were active smokers but no significant differences regarding smoking status were observed between the two groups (*p* = 0.7).

**Table 4:** Baseline characteristics: treated group, control group, and placebo group

	Total (*n* = 85)	Treated group (*n* = 30)	Control group (*n* = 24)	Placebo group (*n* = 31)	*p*-value
Age (years)	52 (44–62)	51.5 (43.5–59.5)	52.8 (44.8–60.8)	52.2 (44.2–60.2)	0.571
Sex					
Male	60	21	17	22	0.704
Female	25	9	7	9	0.85
Type of diabetes					
Type II	63	22	17	24	0.955
Diabetic nephropathy					
Yes	13	5	4	4	0.926
No	72	25	20	27	0.581
Diabetic neuropathy					
Yes	41	14	14	13	0.975
No	44	16	10	18	0.306
Diabetic retinopathy					
Yes	9	3	2	4	0.67
No	76	27	22	27	0.657
Intervention prior to randomization					
Yes	35	12	10	13	0.99
No	50	18	14	18	0.004
Smoke					
Yes	36	13	10	13	0.77
No	49	17	14	18	0.76
HBA1C %	8.9 (6.2–14.2)	12.4	9.7	10.8	0.845
D.M. duration, years	23 (5–41)	40	26	31	0.21
ABI	1.03 (0.4–1.7)	1.6	1.16	1.35	0.965
NSS	0 (0–6)	3	4	3	0.904
NDS	7 (0–9)	4	6	5	0.81
Type of ulcer					
Neuropathic ulcer	43	15	12	16	0.739
Ischemic ulcer	23	8	6	9	0.736
Neuropathic/ischemic ulcer	12	4	3	5	0.77
Ulcer after amputation	7	2	2	3	0.866

Data are presented as mean or number (percentage)

*DM* diabetes mellitus, *HbA1c* hemoglobin A1c, *ABI:*Ankle Brachial Index, *NSS* Neurological Symptom Score, *NDS* Neuropathy Disability Score

There was no difference between the groups in terms of baseline Ankle Brachial Index (ABI) (*p* = 0.96), Neurological Symptom Score (NSS) (*p* = 0.904), or Neuropathy Disability Score (NDS) (*p* = 0.81) in Table 4.

Approximately 42% of all participants were active smokers but no significant differences regarding smoking status were observed between the three groups (*p* = 0.76).

### Outcome of treatment

**Fig. 7:**
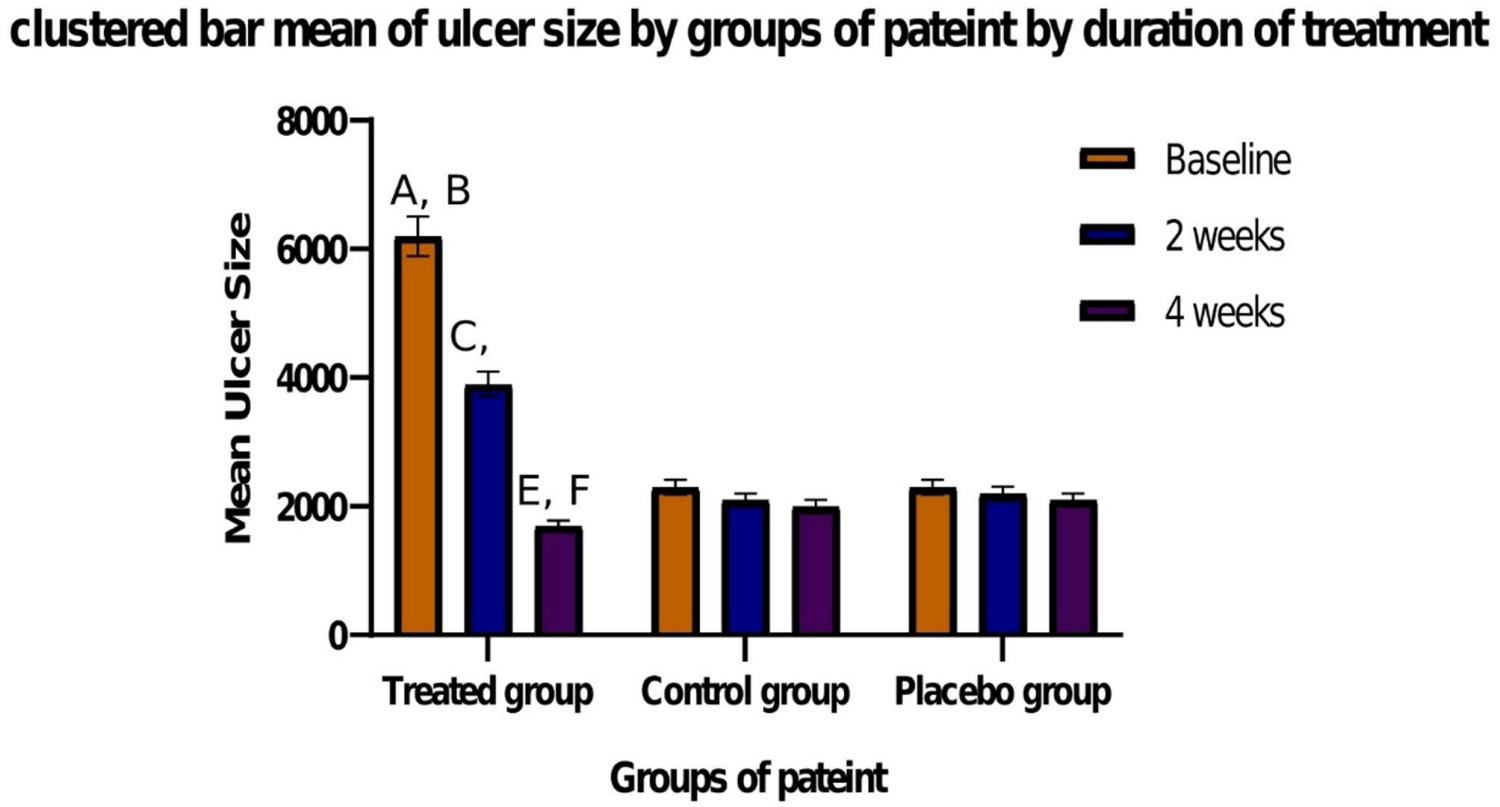
Graph representing the ulcer healing in different study groups (Treated group *n* = 30, Control group *n* = 24, Placebo group *n* = 31). The treatment group showed a significantly larger wound area compared to the control and placebo groups. **A** Represent a high significance difference between the mean ulcer size base line and the mean ulcer size in the control group (*p* > 0.01). **B** Represent a high significance difference between the mean ulcer size base line and the mean ulcer size in the placebo group (*p* > 0.01). **C** Represent a high significance difference between the mean ulcer sizes 2 weeks after treatment and the mean ulcer size in the control group (*p* > 0.01). **D** Represent a high significance difference between the mean ulcer sizes 2 weeks after treatment and the mean ulcer size in the placebo group (*p* > 0.01). **E** Represent a significant difference between the mean ulcer sizes 4 weeks after treatment and the mean ulcer size in the control group (*p* > 0.05). **F** Represent a significant difference between the mean ulcer sizes 4 weeks after treatment and the mean ulcer size in the placebo group (*p* > 0.05)

In comparison to patients in the control and placebo groups in Fig. 7, patients in the treatment group had a considerably larger wound area (*p* = 0.001), indicating a significant difference in ulcer size across the three groups.

At baseline, the treatment group’s median ulcer area was 6 cm^2^ (IQR 5.2–8.5), the control groups were 2 cm^2^, and the placebo groups were 2.7 cm^2^. When compared to baseline at 2 and 4 weeks, the treatment group’s ulcer area and the control group’s ulcer size were significantly reduced. Patients in both groups showed a substantial decrease in ulcer size at 6 weeks after therapy when compared to baseline, however, patients in the placebo group showed no discernible decrease in ulcer size.

Patients in the treated group showed a substantial decrease in ulcer size at 2 and 4 months after therapy, as compared to baseline. In a similar vein, controls showed a notable decrease in ulcer size 2 and 4 months after therapy.

### Complete healing

The mean time for complete healing was 6 weeks (range: 4–8 week) for the treated group and 20 weeks (range: 12–2 8 week) for the controls. At the end of the study, 53 patients (62%) had achieved complete healing, with the percentage of patients in the treated group achieving complete healing being significantly higher than that in the control group.

### Safety-Adverse events table

Before the trial was finished, one patient in the placebo group passed away. Neither the therapy nor the existence of diabetic foot ulcers was linked to the fatalities. Three patients in the treatment group and five in the control group left the trial before it was finished. At the 16-week mark, adverse events were discovered in the patients. The rate of adverse events did not significantly differ between the two groups.

**Fig. 8:**
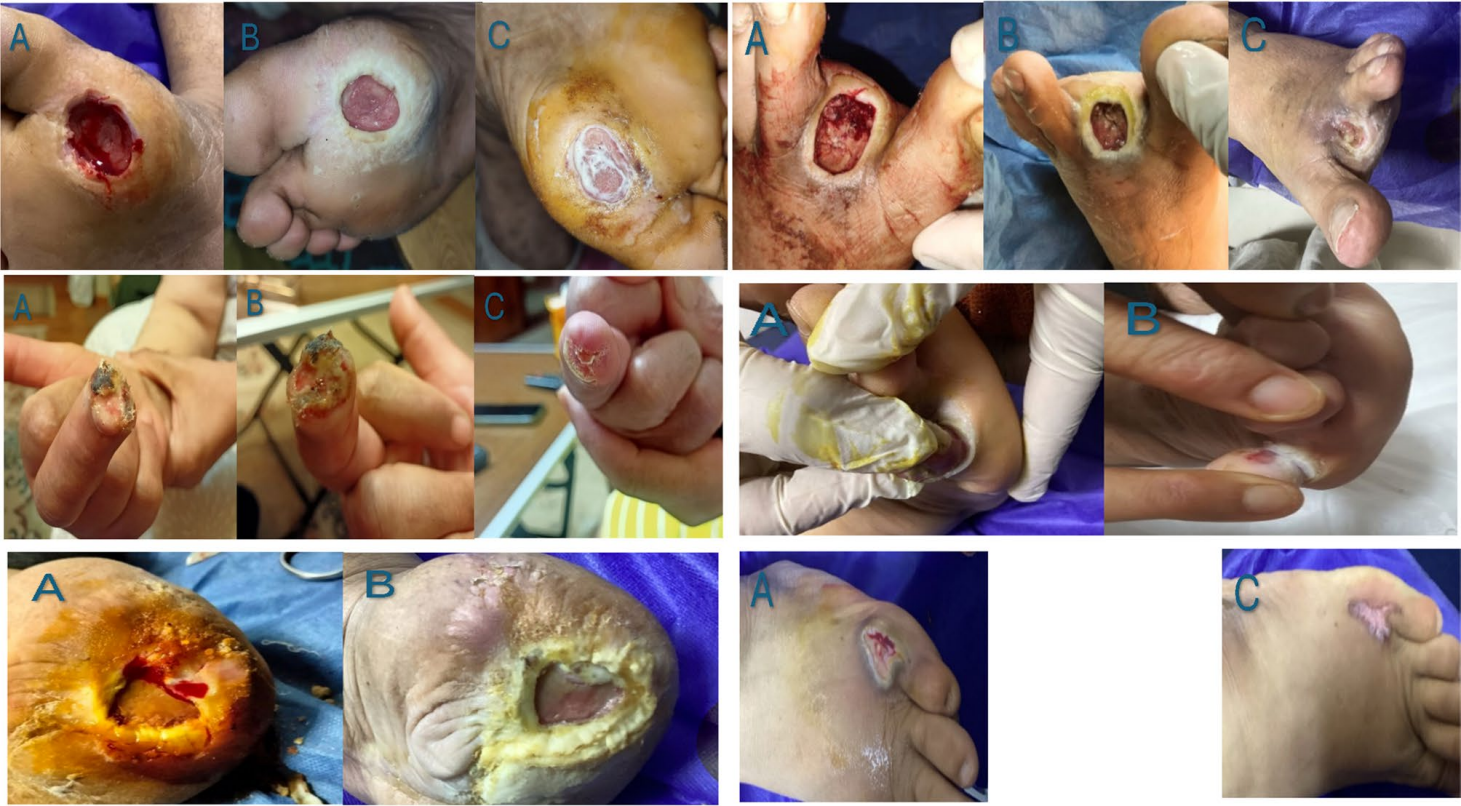
Representative images showing progressive healing of diabetic foot ulcers treated with WJ-MSC-derived exosomes gel. **A** Baseline image of the ulcer before treatment, showing initial wound size and severity. **B** Week 2 post-treatment, demonstrating noticeable reduction in wound size and improved tissue appearance. **C** Week 4 post-treatment, highlighting significant wound closure and advanced healing, indicating the potential efficacy of the exosome gel in promoting tissue regeneration and repair

Two patients (6%) in the treated group developed wound infections compared to 7(29%) patients in the control group, one patient (3%) presented osteomyelitis in the treated group, and two (8%) in the controls (*p* > 0.999). Table 5. five adverse events were documented, none of which were a direct result of WJ-MSC-derived exosome gel as reported by clinicians. Commonly reported adverse events included infection reported in 2 cases (6%), fever 2 (6%), and blisters 3 (10%) (Fig. 8).

**Table 5:** Wound closure outcomes, WJ-MSC usage summary, and adverse events. *n* = 30

Complete wound closure at 12 weeks	
Number of wounds, n (%)	30 (100%)
Percent area reduction at 12 weeks (%)	
Mean ± SD	95 ± 5
Time to complete wound closure (weeks)	
Mean ± SD	6 ± 2
Number of gel applications over 12 weeks	
Mean ± SD	4 ± 4
Adverse effects	
Fever	2 (6%)
Infection	2 (6%)
Osteomyelitis	1 (3%)
Blisters	3 (10%)
New ulcer with purulent discharge	1(3%)

Data are reported as n (%). None of the adverse events were due to the WJ- MSC usage

## Discussion

A serious side effect of diabetes mellitus, diabetic foot ulcers (DFUs) greatly increase morbidity, medical expenses, and lower quality of life [38] These chronic wounds have complex pathology. Due to a combination of neuropathy, poor circulation, and impaired immune responses, making them particularly difficult to manage [39]. Conventional treatments, including debridement, infection control, offloading, and advanced wound dressings, often fail to achieve complete wound closure, frequently leading to severe outcomes such as amputations [40].

The therapeutic potential of mesenchymal stem cell-derived exosomes (MSC-Exos), especially those formed from Wharton’s jelly, in DFU healing has been brought to light by recent studies [12]. MSC-Exos efficiently modulate immunological responses, enhance angiogenesis, and promote tissue regeneration while retaining the regenerative and immunomodulatory qualities of their parent cells. Exosomes’ bilayer membrane shields their contents, guaranteeing stability and functioning when used therapeutically [41] Human umbilical cord-derived MSCs (hUC-MSCs) exhibit strong targeting specificity in diabetic foot ulcer models, precisely localizing to the injured tissue [42].

A significant factor in the pathophysiology of DFU is diabetic neuropathy, which affects the autonomic, motor, and sensory nerves. Autonomic dysfunction affects circulation, motor neuropathy causes foot abnormalities, and sensory neuropathy causes pain or loss of feeling. It has been demonstrated that MSC-Exos stimulate signaling pathways such PI3K/Akt, ERK, and STAT3, which in turn stimulate the production of neurotrophic factors like stromal cell-derived factor-1 (SDF-1), insulin-like growth factor-1 (IGF-1), and nerve growth factor (NGF). Moreover, exosomal miRNAs, including as miR-199b, miR-218, and miR-148a, are essential for vascular regeneration, neuronal differentiation, and proliferation [43] particularly WJ-MSC -exosomes as proved by previous studies could transfer brain-derived neurotrophic factor (BDNF) to retinal neurons and activate the BDNF-TrkB pathway to enhance high glucose (HG)-stimulated neuronal cell viability and inhibit its apoptosis [44].

Both macroangiopathy and microangiopathy contribute to impaired angiogenesis in diabetes, which results in inadequate tissue perfusion [45] By paracrinely releasing angiogenic factors such as vascular endothelial growth factor (VEGF), basic fibroblast growth factor (BFGF), hypoxia-inducible factor-1 (HIF-1), and epidermal growth factor (EGF), MSC-Exos promote neovascularization [37]. These elements promote vascular regeneration and wound healing by enhancing the formation of extracellular matrix (ECM), preventing apoptosis, and supporting endothelial cell survival [46, 47].

Diabetic individuals are more susceptible to infections due to compromised immune defenses [42, 48, 49] MSC-Exos contributes to antimicrobial activity by improving macrophage function, enhancing neutrophil activity, and promoting bacterial clearance. Moreover, MSC-Exos pretreated with TNF-α/hypoxia has demonstrated efficacy in reducing bacterial burden and colonization in infected DFUs. Additionally, they attenuate oxidative stress-induced damage by modulating NOX1 and NOX expression, reducing reactive oxygen species (ROS) levels, and promoting antioxidant responses [49].

It was discovered that continuous hyperglycemia pushes macrophages toward a sustained M1 phenotype, worsening chronic inflammation and hindering wound healing. Human umbilical cord mesenchymal stem cells (hUC-MSCs) have been found to alter macrophage polarization, therefore relieving pancreatic dysfunction in type 2 diabetes, highlighting their potential therapeutic involvement in macrophage-mediated tissue repair [50, 51].

62% of the patients in our research recovered completely by the conclusion of the trial, and the treated group had a far larger percentage of patients who recovered completely than the control group. The treated group’s median duration to full recovery was 6 weeks.

The treated group exhibited a significant decrease in ulcer area at two and 4 weeks (the treated group’s baseline median ulcer size surface area was 6 cm^2^, after 2 weeks it was 4 cm^2^, and after 4 weeks it was 2.7 cm^2^). Patients in both groups also showed a significant decrease in ulcer size at 6 weeks after treatment compared to baseline.

Patients in the treated group showed a substantial decrease in ulcer size at 2, to 4 months after therapy, as compared to baseline. At 2 and 4 months after therapy, controls also showed a substantial decrease in ulcer size.

The therapeutic promise of MSC-Exos is evident in their demonstrated capacity to enhance angiogenesis, regulate immune responses, and promote extracellular matrix remodeling, as supported by this study and prior research [52]. However, while the reduction in ulcer size at multiple time points is encouraging, it is important to emphasize that these findings represent an early phase of research. Additional large-scale clinical trials are necessary to confirm these outcomes, optimize dosing strategies, and establish safety profiles for clinical use. These efforts will help ensure that the results can be consistently reproduced in diverse patient populations.

These findings align with existing research, such as in a study by Yu et al. [16] they demonstrated that WJ-MSC MSC-derived exosomes exhibited a higher abundance of wound-healing-associated proteins and evaluated their effects on keratinocyte proliferation, using various concentrations of exosomes that were applied at different times. The increased amount of fibrinogen proteins, such as fibrinogen alpha (FGA) and fibrinogen beta (FGB), in Wharton’s jelly-derived MSC (WJ-MSC) exosomes was associated with their superior proliferative impact on keratinocytes when compared to AD-MSC exosomes.

Similarly, Zhang et al. [53] discovered that combining human umbilical cord MSC-derived exosomes (hUCMSC-exos) with Pluronic F-127 (PF-127) hydrogel significantly enhanced diabetic wound healing. This innovative approach accelerated wound closure, increased CD31 and Ki67 expression, promoted granulation tissue regeneration, and upregulated key growth factors, including vascular endothelial growth factor (VEGF) and transforming growth factor beta-1 (TGFβ-1) [54].

Another study from Yan et al. [49] showed that Human umbilical cord mesenchymal stem cells (HUCMSCs) derived exosomes significantly attenuated oxidative stress-induced damage in human umbilical vein endothelial cells (HUVECs) under high-glucose conditions, Mechanistically, elevated glucose levels cause endothelial cell dysfunction and death by triggering the NF-κB signaling pathway, which impairs tissue-level angiogenesis [55]. According to their results, HUCMSCs improve endothelial function and proliferation, partly via reducing phosphorylated IκB-α and NF-κB through exosome-derived miR-146a, which restores the function of vascular endothelial cells [56].

Finally, one of the main benefits of WJ-MSC exosomes relates to the extracellular matrix (ECM) creation and remodeling, the last stage of wound healing, which is a crucial factor in determining healing duration and scar formation. The function of exosomes in matrix remodeling has been emphasized by recent research. Umbilical cord mesenchymal stem cell-derived exosomes (UCMSC-Exos) have been demonstrated to suppress myofibroblast development and excessive aggregation, hence preventing scarring in vivo through the TGF-β2/Smad2 pathway. These exosomes are abundant in miR-21, miR-23a, miR-125b, and miR-145 [57]. The Wnt4/β-catenin pathway is negatively regulated by the Hippo pathway, which is mostly regulated by Yes-associated protein (YAP), which is phosphorylated by UCMSC-Exos. During the remodeling phase, this process helps balance tissue regeneration and repair while avoiding excessive cell proliferation and collagen deposition [58].

The results underscore the potential of exosomes produced from MSCs as a viable therapeutic strategy for the treatment of diabetic foot ulcers (DFUs).

## Challenges and prospects

Before MSC-Exos can be extensively used to treat diabetes and its consequences, several issues need to be resolved, despite their encouraging promise. The biodistribution and retention of MSC-Exos in target tissues is a major concern. According to studies, only a tiny portion of exosomes delivered systemically stay in the liver and spleen for more than 24 to 48 h, most likely because of excessive vascularization. The processes behind MSC-Exos homing and their long-term therapeutic benefits require more investigation.

The variability of MSC-Exos presents another difficulty. Different tissue-derived exosomes have unique functional and proteomic characteristics, which might affect how well they work as medicines. Exosome composition and activity can also be affected by differences in isolation procedures, storage strategies, and culture circumstances. To guarantee repeatability and maximize therapeutic results, MSC-Exos manufacturing and characterization must be standardized.

Furthermore, there are currently few investigations on other diabetic sequelae such as pancreatic damage and diabetic kidney disease (DKD), with the majority of MSC-Exos uses in diabetes focusing on DFUs. To improve MSC-Exos stability and efficacy in vivo, tailored and sustained-release delivery system advancements are required.

Lastly, more clinical research is necessary to confirm MSC-Exos’ safety and therapeutic potential. MSC-Exos has been shown in preliminary clinical trials to improve renal function and inflammatory responses in chronic kidney disease. New exosome-based treatment approaches will be made possible by extending these investigations to diabetes and its associated problems.

## Conclusion

Our research adds to the increasing amount of data indicating that MSC-Exos is a viable treatment option for DFUs. MSC-Exos provide a multimodal approach to improve wound healing outcomes in diabetes patients by focusing on several pathogenic pathways, such as inflammation, angiogenesis, neuroprotection, infection control, and extracellular matrix remodeling. To maximize their therapeutic effectiveness and provide standardized methods for their clinical application, more extensive clinical trials are necessary.

## Data Availability

All data are available upon request from authors.

## Abbreviations

AD-MSC: Adipose-derived mesenchymal stem cells
Akt: Protein kinase B (PKB)
DFU: Diabetic foot ulcer
DMEM: Dulbecco’s Modified Eagle Medium
ECM: Extracellular matrix
ERK: Extracellular signal-regulated kinase
FGB: Fibrinogen beta chain
FOXP3: Forkhead box P3.
IDO: Indoleamine 2,3-dioxygenase
IkB: Inhibitor of NF-κB
Ki-67: Marker of proliferation Ki-67
MAPK: Mitogen-activated protein kinase
NF-kB: Nuclear factor kappa-light-chain-enhancer of activated B cells
NOX-1: NADPH oxidase 1
PI3K: Phosphoinositide 3-kinase
PTEN: Phosphatase and tensin homolog
STAT 3: Signal transducer and activator of transcription
TEM: Transmission electron microscope
WJ-MSC-Exo: Wharton jelly mesenchymal stem cell exosomes.
